# Defining cooperative roles for colonic microbiota and Muc2 mucin in mediating innate host defense against *Entamoeba histolytica*

**DOI:** 10.1371/journal.ppat.1007466

**Published:** 2018-11-30

**Authors:** Aralia Leon-Coria, Manish Kumar, France Moreau, Kris Chadee

**Affiliations:** Department of Microbiology, Immunology and Infectious Diseases, Snyder Institute for Chronic Diseases, University of Calgary, Calgary, Alberta, Canada; University of Medicine & Dentistry New Jersey, UNITED STATES

## Abstract

Amebiasis is caused by the protozoan parasite *Entamoeba histolytica* (*Eh*), a potentially fatal disease occurring mainly in developing countries. How *Eh* interacts with innate host factors in the gut is poorly understood. *Eh* resides and feed in/on the outer colonic mucus layer and thus share an ecological niche with indigenous microbiota. As gut microbiota regulates innate immune responses, in this study we characterized the cooperative roles that microbiota and the mucus layer play in *Eh*-induced pro-inflammatory responses in the colon. To study this, we used antibiotics treated and non-treated specific pathogen free *Muc2*^*-/-*^ and *Muc2*^*+/+*^ littermates and germ-free mice inoculated with *Eh* in colonic loops as a short infection model. In antibiotic treated *Muc2*^*-/-*^ and *Muc2*^*+/+*^ littermates, *Eh* elicited robust mucus and water secretions, enhanced pro-inflammatory cytokines and chemokine expression with elevated MPO activity and higher pathology scores as compared to the modest response observed in non-antibiotic treated littermates. Host responses were microbiota specific as mucus secretion and pro-inflammatory responses were attenuated following homologous fecal microbial transplants in antibiotic-treated *Muc2*^*+/+*^ quantified by secretion of ^3^H-glucosamine newly synthesized mucin, Muc2 mucin immunostaining and immunohistochemistry. *Eh*-elicited pro-inflammatory responses and suppressed goblet cell transcription factor Math1 as revealed by *in vivo* imaging of *Eh*-colonic loops in Math1^GFP^ mice, and *in vitro* using *Eh*-stimulated LS174T human colonic goblet cells. *Eh* in colonic loops increased bacterial translocation of bioluminescent *E*. *coli* and indigenous bacteria quantified by FISH and quantitative PCR. In germ-free animals, *Eh*-induced mucus/water secretory responses, but acute pro-inflammatory responses and MPO activity were severely impaired, allowing the parasite to bind to and disrupt mucosal epithelial cells. These findings have identified key roles for intestinal microbiota and mucus in regulating innate host defenses against *Eh*, and implicate dysbiosis as a risk factor for amebiasis that leads to exacerbated immune responses to cause life-threatening disease.

## Author summary

*Entamoeba histolytica* (*Eh*) is a human protozoan parasite and the causative agent of amebiasis. Amebic colitis causes cellular destruction of the colonic mucosal layers allowing parasites to disseminate to the liver through blood causing amoebic liver abscess, or to other soft organs like the brain and lungs. *Eh* in the colon shares an environment with resident intestinal microbiota that lives, feeds and multiplies on the mucus layer. Both microbiota and mucus play critical protective roles in innate host defense however, to date, little is known about the interactions between ameba-microbiota and the mucus layers. Here we show that microbial dysbiosis worsen the outcome of *Eh* infection, favoring bacterial translocation, increasing pro-inflammatory responses independently of the mucus layer, as well as regulating mucus release from goblet cells. The absence of microbiota in germ-free mice altered the host from mounting a proper innate immune response towards *Eh* allowing parasites to bind to and disrupt mucosal epithelial cells. These data highlight critical roles for indigenous microbiota in imprinting and educating mucosal innate host defenses critical for host defense against *E*. *histolytica* invasion.

## Introduction

*Entamoeba histolytica* (*Eh*) is a human protozoa parasite that causes the disease amebiasis and that, in 2013, was responsible for approximately 11,300 deaths worldwide [1]. Interestingly, of those individuals infected with the parasite, only 10% develop symptoms of the disease while the rest remain asymptomatic. To date, the variation in disease outcome has not been fully explained, but several studies have suggested that this difference could be due to host´s health conditions as well as immune fitness. More recently, with molecular tools for studying the microbiome, the idea that specific bacteria within the microbiota could modulate *Eh* pathogenesis and predispose to intestinal amebiasis [2] and amebic liver abscess (ALA) [3] has been proposed.

MUC2 is the major secretory mucin in the gastrointestinal tract and forms a barrier between the epithelial cells monolayer and the luminal content that not only consist of nutrients, but is also loaded with potential pathogenic microorganisms. When *Eh* is ingested through contaminated food or water, it colonizes the colonic outer mucus layer, which is rich in a diverse community of bacteria. The human intestine hosts approximately 100 trillion microorganisms [4], mainly bacteria, that form the microbiota that regulates host homeostasis by promoting digestive health as well as stimulating a balanced immune system [4,5]. It is known that *Eh* stimulates goblet cell mucus secretion [6] as an innate host defense mechanism to counter *Eh* adherence to intestinal epithelial surfaces [7]. In disease pathogenesis, *Eh* cysteine proteases cleave MUC2 in the non-glycosylated C-domain, weakening its structure and facilitating *Eh* contact with epithelial cells [8]. Germ-free and gnotobiotic experiments with guinea pigs have established that the presence of gut microbiota is required for *Eh* pathogenicity [9].

In recent studies, the link between microbiota and *Eh* infection in humans has been suggested with varying results depending on location and subjects of the study. A study in India have shown a decrease in mostly butyrate-producing bacteria (*Clostridium coccoides*, *C*. *leptum*, *Eubacterium*, *Lactobacillus*, *Bacteroides*) and an increase in *Bifidobacterium* in stool samples from *Eh* positive patients [10]. More recently, a study in Cameroon found higher α-diversity and a decrease in β-diversity in individuals positive for *Eh* infection with an increase in members of the Clostridiales Ruminococcaceae family and a decrease in *Prevotella copri* [11]. The decrease in *P*. *copri* was confirmed in a longitudinal study done in children living in a slum in Bangladesh and the presence of this bacteria was correlated with higher incidence of diarrhea when compared with asymptomatic *Eh*-positive children [12]. Analysis of bacterial diversity in ALA patients was not able to significantly relate its incidence with any specific bacteria, however, co-infection with bacteria was present in most of the ALA samples, with a notable higher abundance of *Klebsiella* [3]. Various studies [2,13–15] have demonstrated that microbiota can significantly alter the outcomes of different protozoan infections, however mechanisms underlying these relationship remain poorly understood.

In this study, we explored the distinct contributions of microbiota and the Muc2 mucus barrier in *Eh*-induced innate and pro-inflammatory responses critical in disease pathogenesis. Our findings show that indigenous commensal microbiota that colonizes the outer Muc2 mucus layer plays an important role in fortifying innate host defense against *Eh* and that dysbiosis (antibiotic and/or alterations in the mucus layer) renders the colonic epithelium susceptible to *Eh*-induced pro-inflammatory responses and tissue injury.

## Results

### Muc2 deficient and sufficient antibiotic-treated mice elicits an enhanced pro-inflammatory cytokine and chemokine response when inoculated with *E*. *histolytica (Eh)*

**Fig 1:**
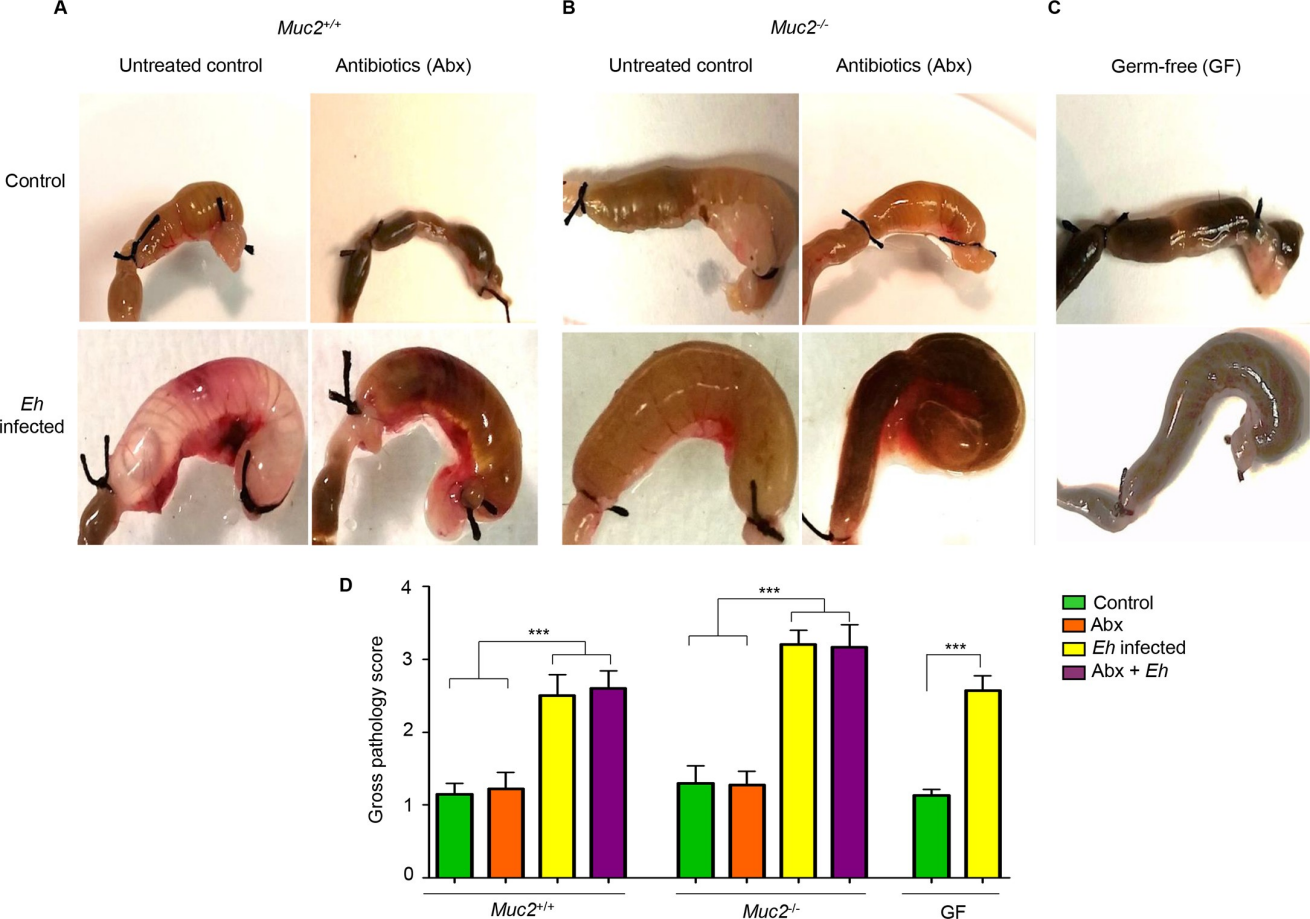
Gross pathology of colonic loops in *Muc2*^*+/+*^, *Muc2*^*-/-*^ and germ-free (GF) mice inoculated with *Entamoeba histolytica* (*Eh)*. *Eh*-colonic loops were performed in antibiotic treated (Abx) and untreated *Muc2*^+/+^ and *Muc2*^-/-^ littermates and in GF mice. Gross pathology was measured and scored among treatments as indicated in Material and Methods. (A) *Eh* inoculated in *Muc2*^+/+^ colons elicited extensive mucoid secretions with bloating. (B) *Eh* in *Muc2*^-/-^ mice showed a similar ballooning effect with abundant watery secretion under intense pressure. (C) Colonic loops in GF mice evoked a less robust watery/mucoid secretions with less ballooning as *Muc2* genotypes. (D) Gross pathology score of colons. Note no differences in gross pathology were observed among *Eh*-inoculated groups compared to homologous controls. n = 6, *** P < 0.001.

To quantify the distinct roles of indigenous microbiota and/or the presence of an intact Muc2 mucus layer in *Eh*-induced innate host responses in closed colonic loops [16], *Muc2*^+/+^ and *Muc2*^-/-^ littermates were pre-treated or not with a broad spectrum antibiotic (Abx) cocktail and compared with germ-free mice (GF). *Eh* inoculated in colonic loops in *Muc2*^+/+^ presented with watery and mucoid secretions under intense pressure and bloating (gas bubble formation). Abx-treated animals showed similar responses but also presented with bloody secretions (**Fig 1A**) as compared to PBS-inoculated controls. *Muc2*^-/-^ littermates in the absence of a mucus layer showed similar ballooning effects with abundant watery secretions (**Fig 1B**). Similarly, colonic loops in GF mice presented with ballooning mucoid secretions under intense pressure (**Fig 1C**). Overall, no significant differences were observed in gross pathology scores between the Muc2 genotypes and GF mice inoculated with *Eh*. Similarly, Abx treatments did not modify gross pathology scores (**Fig 1D**).

**Fig 2:**
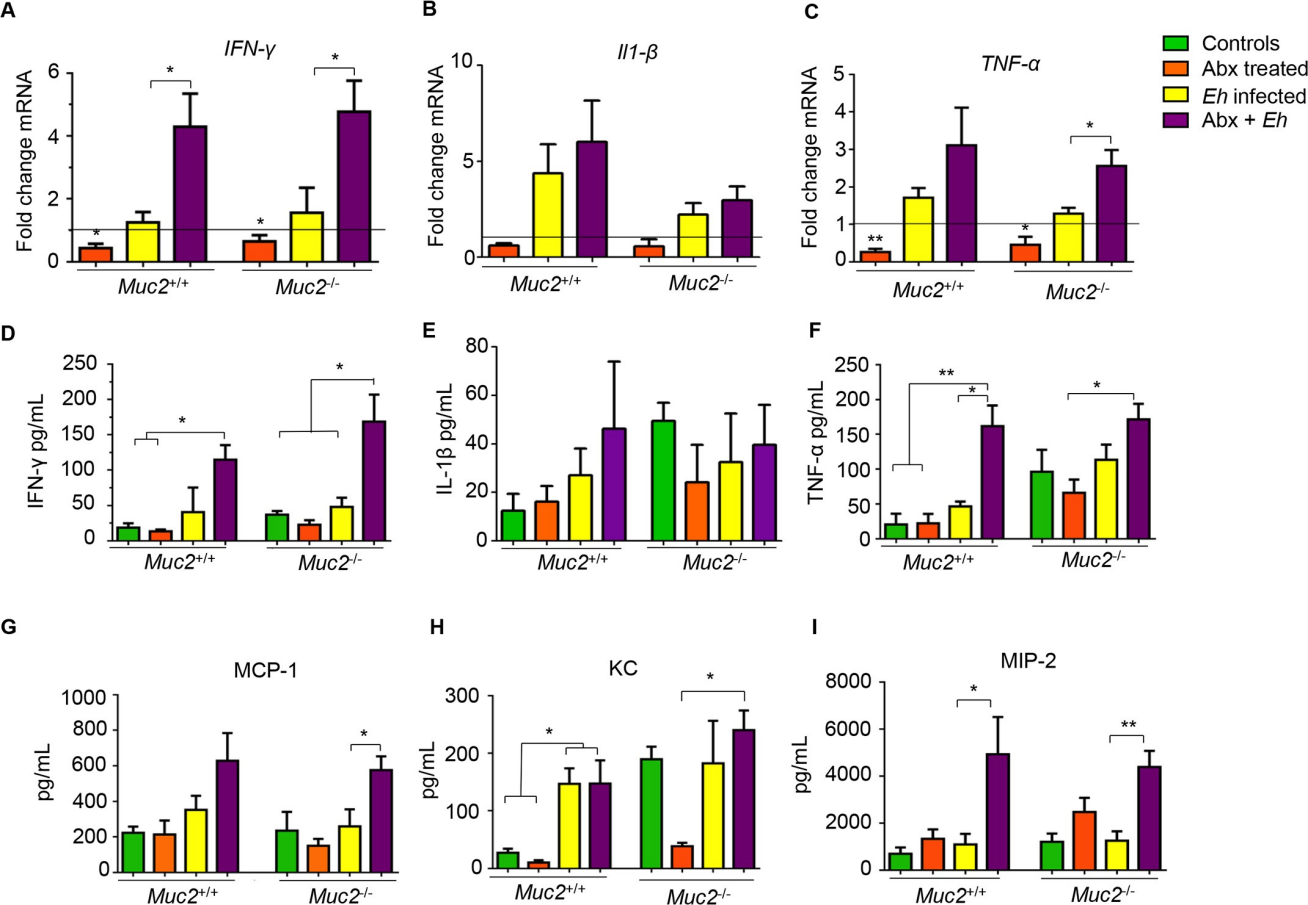
*Entamoeba histolytica* (*Eh*) infection in antibiotic treated mice exacerbates pro-inflammatory responses in *Muc2*^*+/+*^ and *Muc2*^*-/-*^ littermates. (A-C) *Muc2*^*+/+*^ and *Muc2*^*-/-*^ littermates pro-inflammatory cytokines mRNA expression in colonic tissue were measured by qPCR and compared with their corresponding untreated control (line). (D-F) Colonic luminal pro-inflammatory cytokines and chemokines (G-I) protein levels were measured using a mouse cytokine/chemokine array. n = 6. *P < 0.05, **P < 0.01.

To determine whether Abx treatment altered *Eh*-induced host inflammatory responses in Muc2 genotypes, colonic tissues and luminal contents were analyzed for pro-inflammatory cytokines and chemokines. Interestingly, following Abx treatment, basal IFN-γ and TNF-α pro-inflammatory cytokine expression decreased in colonic tissues in both *Muc2*^*+/+*^ and *Muc2*^*-/-*^ littermates as compared to untreated controls (**Fig 2A–2C**). However, as predicted, *Eh*-inoculated colonic loops showed increased pro-inflammatory cytokines expression regardless of the presence or absence of a mucus layer. Surprisingly however, Abx-treated mice inoculated with *Eh* showed significant increase in the pro-inflammatory cytokines IFN-γ and TNF-α mRNA expression as compared with non Abx-treated *Eh* inoculated littermates (**Fig 2A–2C**). A similar increase in IFN-γ and TNF-α protein levels were noted in the luminal contents of Abx-treated *Muc2*^+/+^ and *Muc2*^-/-^ littermates inoculated with *Eh* compared as compared to none Abx-treated controls (**Fig 2D–2F**). Likewise, chemokine levels (MCP-1, KC and MIP-2) in the luminal contents of Abx-treated animals inoculated with *Eh* were significantly increased compared with non Abx-treated controls (**Fig 2G–2I**). Several other cytokines and chemokines were assessed by multiplex, however their differences were not significant. These results clearly show that a dysbiotic state induced by Abx-treatment predisposes the host for robust pro-inflammatory responses toward *Eh* regardless of the presence or absence of a functional mucus barrier.

### *Eh* in antibiotic-treated mice evokes a robust mucus secretagogue response

**Fig 3:**
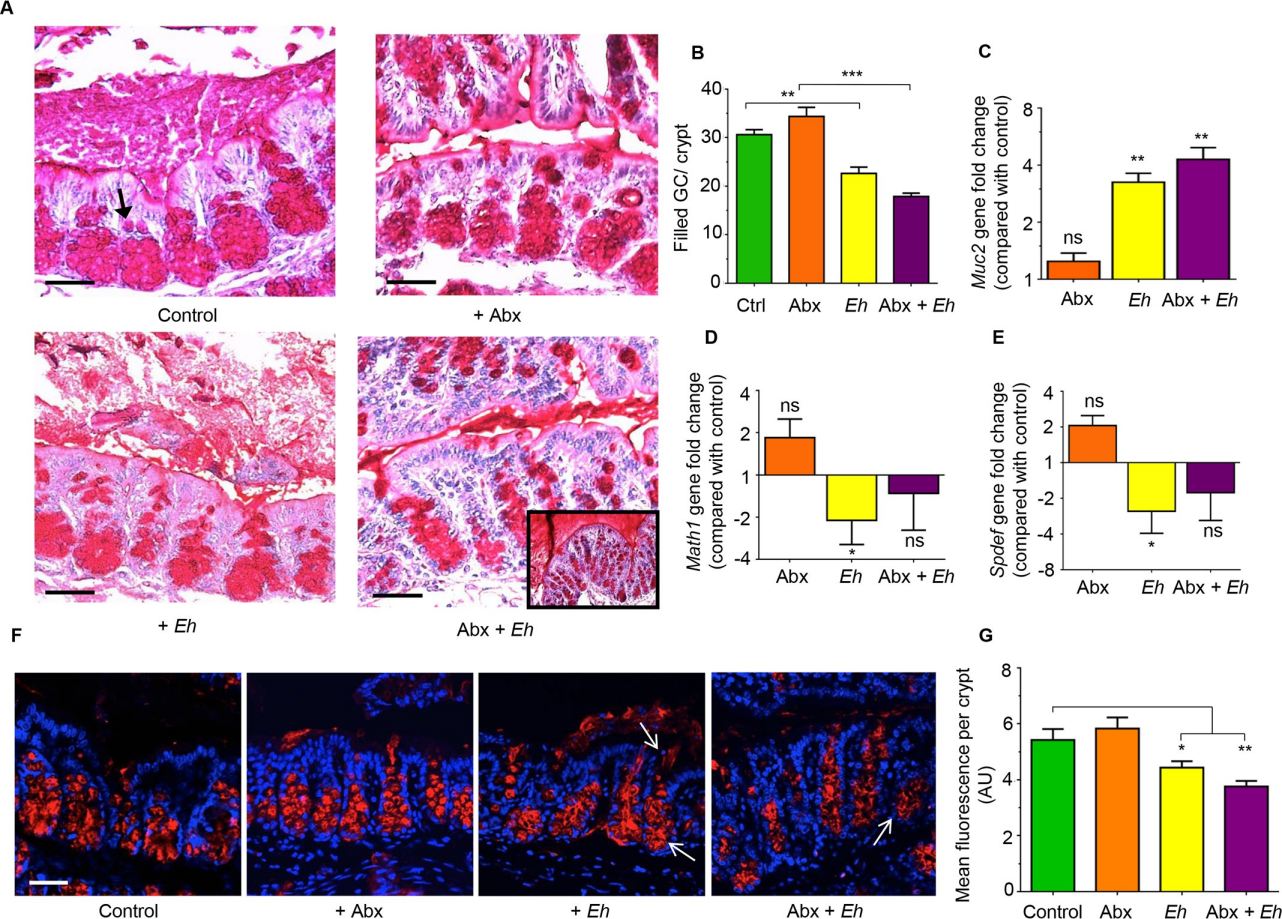
*Entamoeba histolytica* (*Eh*) in antibiotic-treated mice triggers robust mucus and Muc2 mRNA expression. (A) Colonic loops from *Muc2*^+/+^ mice were fixed in Carnoy’s solution to preserve the mucus layers and stained with Periodic acid Schiff (PAS) reagent to visualize secreted and goblet cell mucus. Top left untreated control proximal colon with densely packed pear-shaped collection of goblet cells in the crypts filled with PAS^+^ material (arrow). Top right is the Abx- treated group showing similar morphology to the controls. Bottom left is *Eh*-inoculated colonic loops with secreted mucus is a watery exudate and less PAS+ cells in the crypts. Bottom right is the Abx treated mice inoculated with *Eh* showing fewer filled PAS+ cells in the crypts and with a dense mucus plugs on the surface of the mucosa (inset). (B) Quantification on the number of filled goblet cells per crypt. (C) Gene expression for *Muc2*, (D) *Math1* and (E) *Spdef*. (F) Confocal microscopy using Muc2 specific antibody (red) and counterstained with DAPI (blue) to visualize Muc2 filled goblet cells in sections for A above. (G) Fluorescence quantification of Muc2 signal per crypt from images in F was analyzed using ImageJ software. Scale bar = 50 μm. n = 6. *P < 0.05, **P < 0.01, ***P < 0.001.

As an intact Muc2 mucus layer and hyper secretion of mucus are critical determinants of innate host defense against *Eh* [17], we determine if Abx treatment in *Muc2*^*+/+*^ littermates affected mucin biosynthesis and secretion. This was monitored in colonic tissues stained with periodic acid Schiff (PAS) reagent to visualize the mucus layer and filled goblet cells, transcription factors for goblet cell lineage and immunostaining for Muc2 mucin within goblet cells. *Muc2*^+/+^ controls and Abx-treated animals showed normal colonic architecture with numerous goblet cells (arrow) filled with PAS^+^ material (**Fig 3A**-top panel). However, when inoculated with *Eh*, *Muc2*^+/+^ showed prompt robust mucus secretory response (**Fig 3A**-bottom left) with coalescence of mucin granules and mucus streaming out in the lumen. In Abx-treated animals, *Eh* elicited enhanced mucus secretion (**Fig 3A**-bottom right) forming a thick mucus plug over the mucosal surface (**Fig 3A**-bottom right inset). Mucus secretagogue effects evoked by *Eh* correlated well with the number of filled goblet cells per crypt. In particular, there was a significant decrease in the number of filled goblet cells in control and Abx-treated mice inoculated with *Eh* as compared with controls that received PBS (**Fig 3B**). While Abx treatment alone did not affect basal *Muc2* gene expression, in response to *Eh*, both controls and Abx treated animals showed significant upregulation of *Muc2* mRNA expression (**Fig 3C**). Interestingly, Abx treatment had no effect on basal transcription for the secretory cell lineage *Math1*, however in response to *Eh*, *Math1* gene expression was significantly decreased but had no effect in Abx-treated animals (**Fig 3D**). As *Math1* affects all secretory lineage we analyzed *Spdef* expression [SAM pointed domain containing ETS transcription Factor (SPDEF)], the transcription factor that is critical for terminal goblet cell differentiation [18] and observed the same trend (**Fig 3E**). Based on the decreased number of filled goblet cells in *Eh*-inoculated animals with a corresponding increase in *Muc2* gene expression, immunostaining of colonic loops was done to visualize Muc2 mature mucin granules within goblet cells. Even though a similar pattern to the PAS staining was observed with the Muc2 antibody, immunostaining revealed mucin granule-granule coalescence and mucus streaming from goblet cells in the deep crypts in response to *Eh* (**Fig 3F** arrows). There was a paucity of filled goblet cells with mucin in Abx + *Eh* inoculated animals (**Fig 3F** arrows) demonstrating intense mucus secretion with cavitation and/or mucus depleted goblet cells (**Fig 3G**).

**Fig 4:**
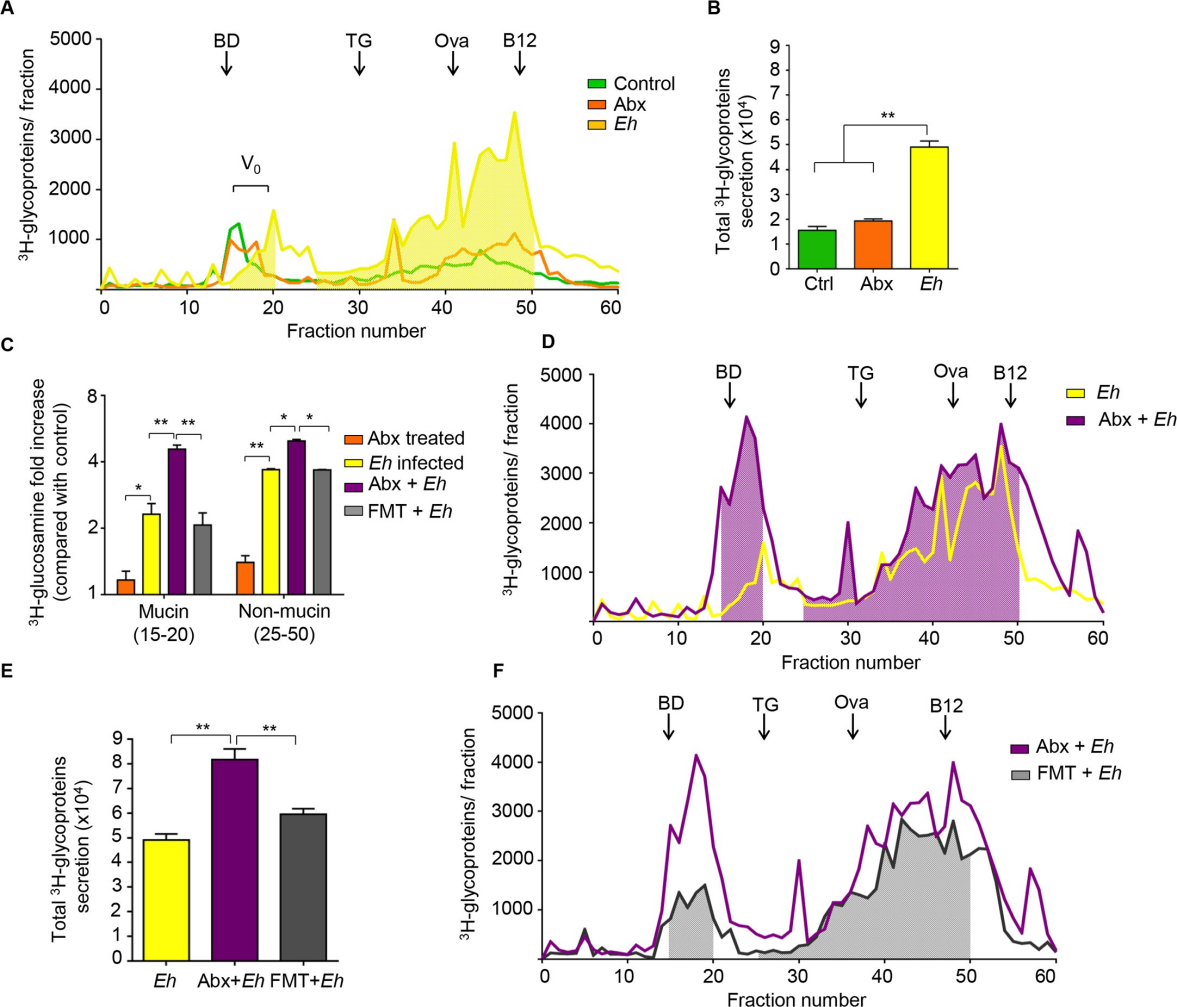
Reduction in bacterial load with antibiotics increases *Eh*-induced colonic mucus and non-mucus secretions. To quantify the release of newly synthesized mucus, mucin was metabolically labeled with ^3^H-glucosamine that incorporates into mucin galactose and N-acetylgalactosamine glycan residues. (A, D, F) In response to *Eh* in closed colonic loops, secreted ^3^H-labeled glycoproteins were fractionated on a Sepharose 4B column to separate high molecular weight mucin (Vo; fractions 15–20) from non-mucin glycoproteins (fractions 25–50). (B, E) Total ^3^H- counts (CPM) released constitutively in control, Abx-treated and fecal microbial transplant (FMT) animals in response to *Eh*. (C) Fold change in ^3^H-mucin V_0_ mucin and non-mucin release among the treated groups as shown in the legend. The column was equilibrated with BD: blue dextran (2,000 kilodaltons), TG: thyroglobulin (660 kilodaltons), Ova: chicken ovalbumin (42.7 kilodaltons), B12: Vitamin B12 (1.4 kilodaltons). n = 8, *P < 0.05, **P < 0.01.

To quantify mucin and none mucin glycoprotein secretions, mucus in *Muc2*^+/+^ littermates were metabolically label with ^3^H-glucosamine that incorporates into galactose, N-acetylgalactosamine and N-acetylglucosamine glycans into newly synthesized mucin. The ^3^H-labeled glycoproteins secreted in response to *Eh* were then fractionated into high molecular weight V_o_ mucin and non-mucin components by Sepharose 4B column chromatography (**Fig 4A**) [6]. Abx treatment had no effect on constitutive mucin or non-mucin glycoproteins secreted in the colon as compared with untreated control (**Fig 4A, 4B and 4C orange panels**). Consistent with previous studies [16], *Eh* significantly stimulated not only V_0_ mucin but also non-mucin glycoprotein secretions (**Fig 4A, 4B and 4C yellow panels**). Surprisingly, Abx-treated mice inoculated with *Eh* (Abx + *Eh*) showed enhanced secretions of both mucin and non-mucin glycoproteins (**Fig 4C, 4D and 4E purple panels**) as compared to animals that were not treated with Abx but inoculated with *Eh* (*Eh* infected group). To exclude the possibility that the enhanced mucus secretory effect was due to the Abx, animals received fecal microbial transplantation (FMT) with their own microbiota following Abx treatment and then inoculated with *Eh*. Remarkably, FMT normalized both ^3^H-mucin and non-mucin glycoproteins secretions equivalent of animals inoculated with *Eh* that did not receive Abx (**Fig 4C, 4E and 4F gray**). Taken together, these results clearly indicate that dysbiotic microbiota provokes enhanced mucin and non-mucin secretions in response to *Eh*.

### *Eh* supresses Math1 transcriptional activity in colonic cells

**Fig 5:**
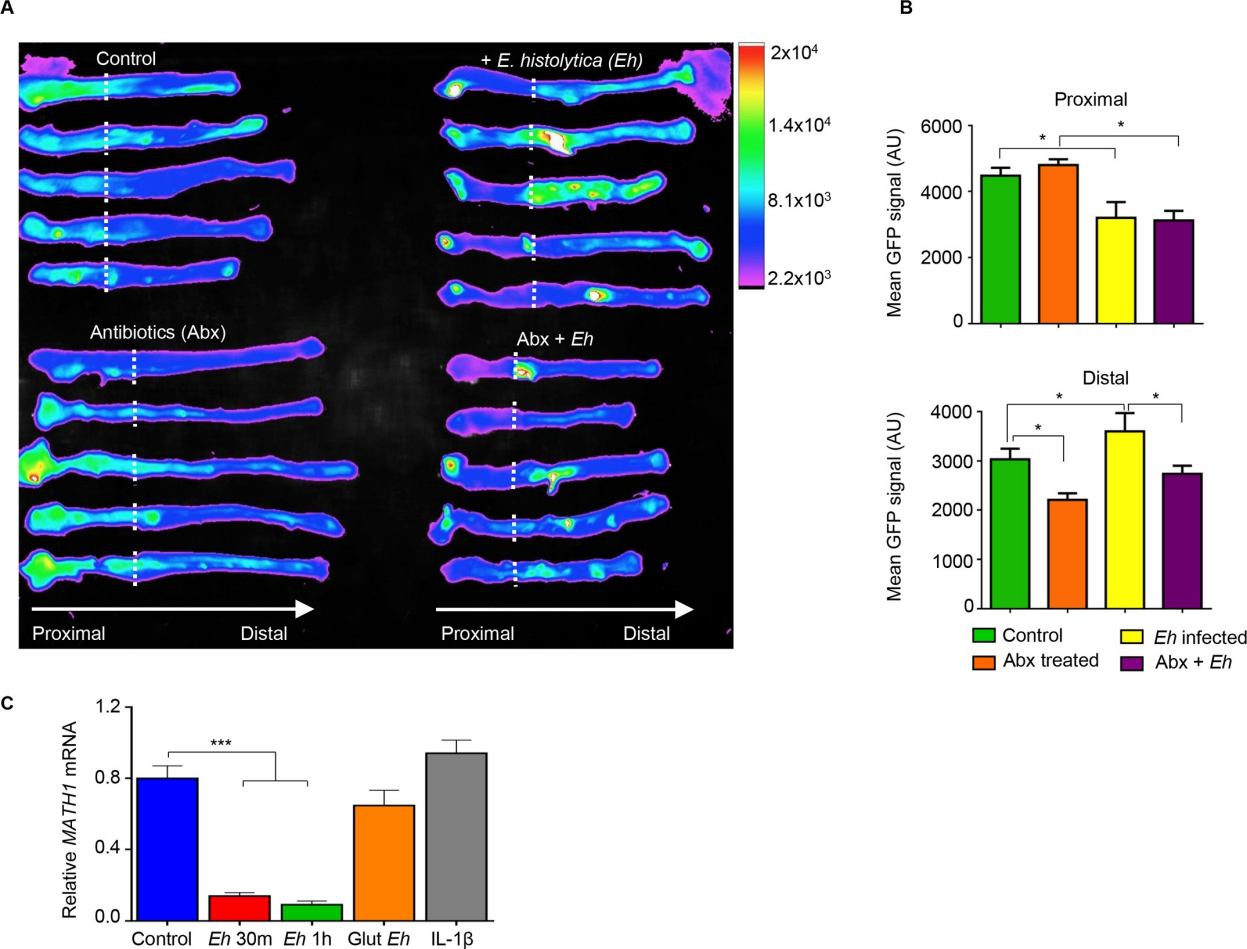
*Entamoeba histolytica* (*Eh*) supresses Math1 mRNA expression in colonic goblet cells *ex vivo* and *in vitro*. (A) Math1 expression heatmap of Math1^GFP^ mice colons, the dotted line indicates the colonic loop ligation. (B) Each colon was divided into proximal and distal regions and GFP signal quantified. (C) Monolayers of LS 174T human goblet cells where either exposed to *Eh* for 30 min (m), 1 h, fixed *Eh* with glutaraldehyde 2.5% (Glut), or pre-treated with IL-1β. mRNA expression levels of transcription factor *MATH1* were measured by qPCR and normalized with *GAPDH* levels. GFP: green fluorescent protein, AU: arbitrary units. n = 6, *P < 0.05, ***P< 0.001.

Based on the differential *Math1* gene expression (**Fig 3D**), we next investigated the fate of the secretory goblet cells in the colon following Abx treatment and in response to *Eh*. To interrogate this, we used Math1^GFP^ mice containing the green fluorescent protein (GFP) reporter for *Math1*-expressing goblet cells. In the colon, *Math1* is expressed in epithelial cells to differentiate into Muc2-producing goblet cells lineage. We have recently used Math1^GFP^ mice to quantify goblet cells by flow cytometry and by imaging to follow the fate of goblet cells in DSS-induced colitis [19]. Basally, Math1^GFP^ activity was higher in the proximal than the distal colon in control animal. However, following Abx treatment, while fluorescence activity in the proximal colon remained unchanged there was a significantly decrease in Math1 activity in the distal colon (**Fig 5A and 5B**). Surprisingly, control mice inoculated with *Eh* showed a significant decrease in Math1^GFP^ activity in the proximal colon (area in direct contact with *Eh*) with a corresponding increase in activity in the distal colon (away from *Eh* interaction). In Abx treated mice, while Math1^GFP^ activity in the proximal colon remained the same as control animals, Math1 activity in both the proximal and distal colon was completely silenced in response to *Eh* (**Fig 5A and 5B**). These results suggest that *Eh* supresses Math1 activity in the proximal colon where *Eh* are contained within the loops and can alter Math1 activity distally.

To determine if the down regulation in Math1 activity was a direct effect from *Eh* or initiated by *Eh*-induced inflammation, LS174T human colonic goblet were inoculated with *Eh* and MATH1 mRNA expression quantified. Within 30 min, *Eh* significantly decreased *MATH1* mRNA expression that remained low up to 1h (**Fig 5C**). Cells stimulated with glutaraldehyde-fixed equivalent numbers of *Eh* had no effect on *MATH1* mRNA expression, suggesting a requirement for live parasites (**Fig 5C**). In acute intestinal amebiasis, IL-1β is one of the most important pro-inflammatory cytokines elicited by cysteine protease 5 (*Eh*CP5) RGD motif ligating host cells integrins [20] and this cytokine had no effect on *MATH1* transcription (**Fig 5C**). These data suggest that *Eh* can also directly inhibit *MATH1* transcription in the absence of bacteria.

### *Eh* infection in the proximal colon increases bacterial translocation

**Fig 6:**
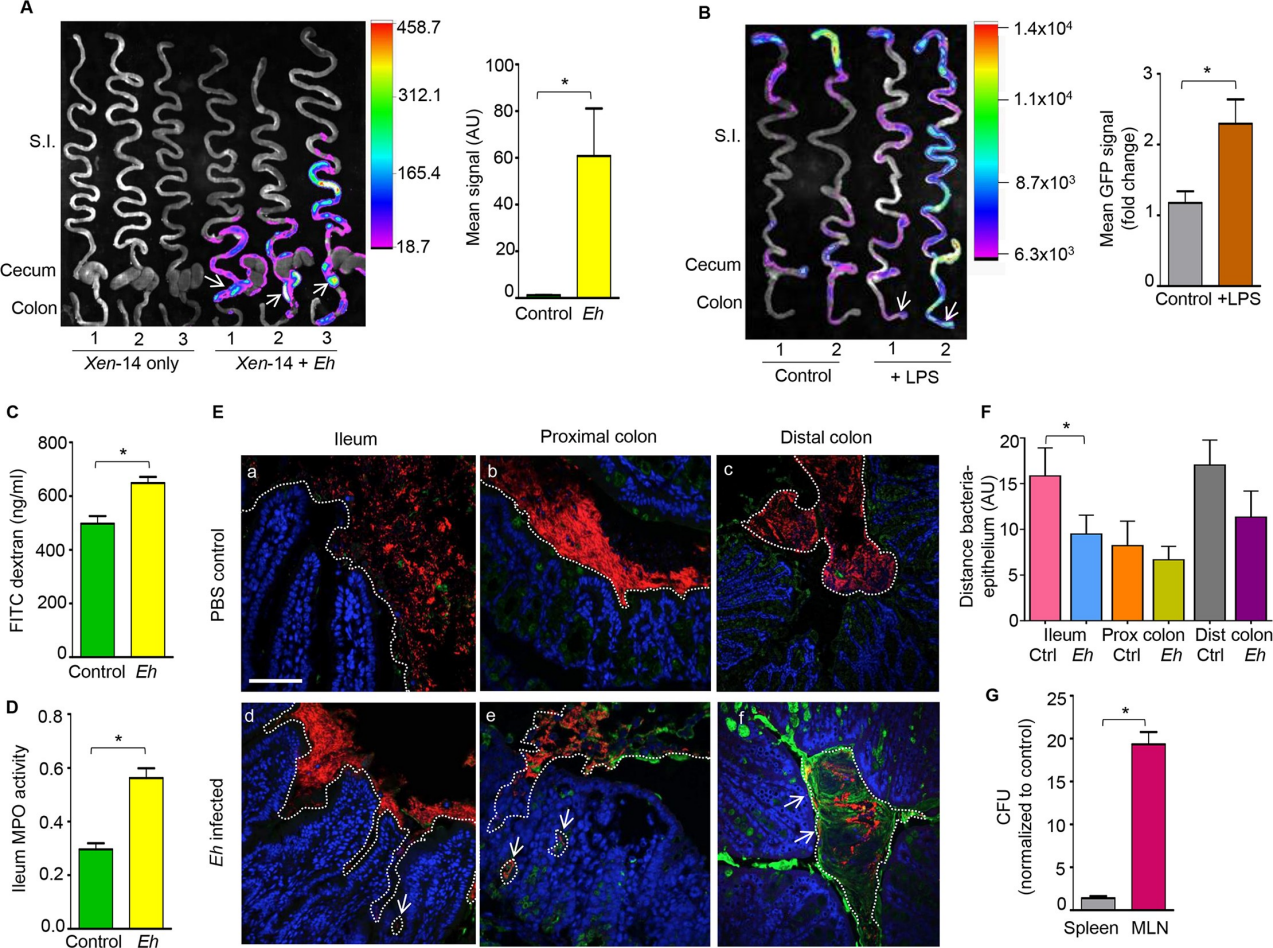
*E*. *histolytica* promotes bacterial translocation. (A) Representative heat map image of the small intestine, cecum and colon of wild type mice inoculated with bioluminescent *E*. *coli XEN* 14 and infected with *Eh* in colonic loops (arrows). The mean bioluminescent signal was quantified and plotted on a histogram. (B) Representative heatmap of Math1 expression in the gastrointestinal tract of Math1^GFP^ mice inoculated with a sub lethal dose of LPS (5mg/kg BW) showing increase Math1 GFP activity in the cecum and distal colon (arrows). Mean GFP signal was quantified and plotted on a histogram. (C) In colonic loops inoculated with *Eh*, intestinal permeability was measured by serum concentration of FITC-dextran (ng/mL) and (D) myeloperoxidase activity in the ileum (absorbance 450nm). (E) Visualization of bacterial localization by florescence *in situ* hybridization (FISH) in the ileum (a, d), proximal colon (b, e), and distal colon (c, f) of *Eh* inoculated loops (d, e, f) and PBS inoculated control loops (a, b, c). Translocated bacteria in response to *Eh* in the ileum, proximal colon and distal colon are show by the arrows. Red: bacteria, blue: enterocytes nuclei, green: mucus. The dotted line delimits area where commensal bacteria were present. Scale bar = 50 μm. (F) Quantification of the distance between bacteria and the epithelium from images in E was analyzed using ImageJ software. (G) Quantification of colony forming units (CFU) per gram present in the spleen and mesenteric lymph nodes (MLN) following *Eh* inoculation in colonic loops. GFP: green fluorescent protein, AU: arbitrary units, Ctrl: control, MPO: myeloperoxidase. n = 6, *P < 0.05.

An important finding was that *Eh* infection in the proximal colon suppressed Math1 expression with a corresponding increased in Math activity in the distal colon (**Fig 5A**). We hypothesized that the dysregulation of Math1 expression could be due to bacterial translocation. To establish if the Math1 activity was associated with increased bacterial translocation, animals were infected with bioluminescent non-pathogenic *E*. *coli XEN*-14 and inoculated with *Eh* in the proximal colon. *Eh* infection elicited significantly increased bioluminescent signals from the proximal/distal colon (arrows shows the site of *Eh* inoculation) up to the ileum and upper small intestine (**Fig 6A**). These results suggest that in response to *Eh*, higher numbers of bacteria came in close contact and/or translocated in the intestinal mucosa that could potentially alter Math1 activity. Thus, to determine if bacterial LPS could regulate Math activity, Math1^GFP^ animals were inoculated with a sublethal dose of LPS (5 mg/Kg BW, intraperitoneally) and observed significantly higher levels of Math1 throughout the full-length of the gastrointestinal tract as compared to controls (**Fig 6B**). This suggests Math1 expression in intestinal goblet cells could be stimulated via inflammation associated with sensing microbial components, (e.g. LPS) which triggers robust mucus secretion to reduce bacterial translocation in the gut. To address this, we first determined if *Eh* inoculated into colonic loops were altering gut permeability that could potentiate bacterial translocation into tissues by assessed intestinal permeability with FITC dextran. As predicted, *Eh* inoculated in colonic loops significant increased intestinal permeability (**Fig 6C**) associated with high levels of MPO activity in the ileum (**Fig 6D**). We have previously demonstrated alterations in tight junction protein expression and loss of epithelial barrier function in the proximal colon inoculated with *Eh* [16,21]. As Xen-14 bacteria (**Fig 6A**) showed increased bioluminescent in the ileum and proximal colon following *Eh*-inoculation, fluorescent *in situ* hybridization (FISH) was done to visualize bacteria translocation. In *Eh*-inoculated colonic loops, most bacteria (red) in the ileum shifted significantly near the villi and deep in the crypts (**Fig 6E-d, Fig 6F arrow**) as compared to PBS inoculated controls (**Fig 6E-a**). In marked contrast, *Eh* inoculation in the proximal colon, disrupted bacteria biofilms into patches that aggregated with mucus strands and/or translocate deep into the crypts and tissues (**Fig 6E-e arrows**) compared to control loops receiving PBS (**Fig 6E-b**). In the distal colon of mice inoculated with *Eh* in the proximal colon there was increased mucus secretion with bacteria close and/or on the surface epithelium (**Fig 6E-f, Fig 6F**) compared to PBS controls (**Fig 6E-c**). We also detected 20-fold higher bacterial counts in the mesenteric lymph nodes (MLNs) using 16S universal primers as compared to controls receiving PBS (**Fig 6G**). Taken together, these data suggest that *Eh*-induced inflammation results in loss of epithelial barrier functions that facilitated commensal bacteria translocation that altered Math1 expressions in the ileum and proximal colon.

### Colonic microbiota is required for development of innate host defense against *Eh*

**Fig 7:**
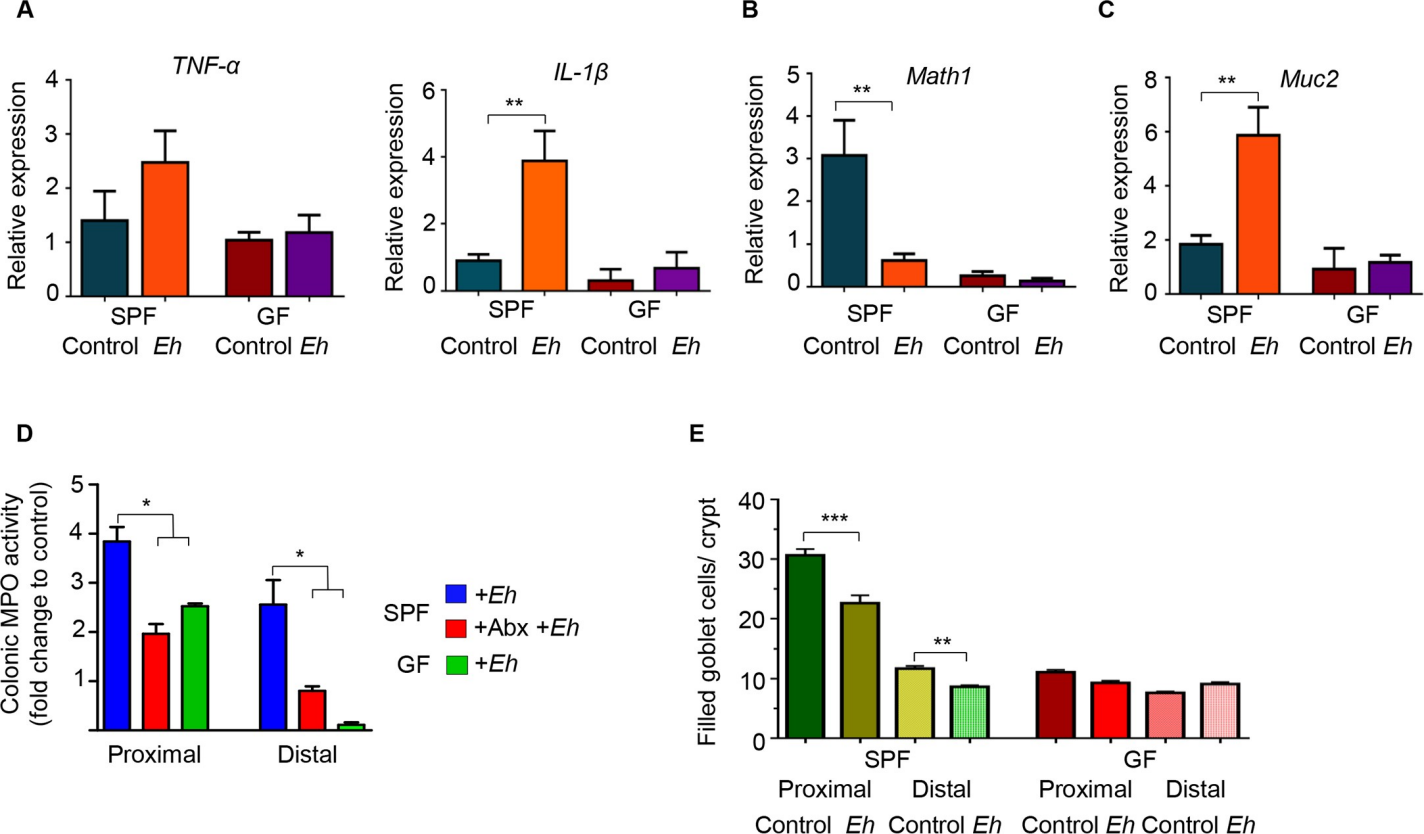
Germ-free mice have a deficient immune response towards *E*. *histolytica*. Amebic colonic loops were performed in germ-free (GF) mice and pro-inflammatory response towards *Eh* was measured and compared with specific pathogen free (SPF) animals. (A) Relative expression of pro-inflammatory cytokines *TNF-α* and *IL-1β* in the proximal colon. (B) Relative expression of the transcription factor *Math1*, and (C) *Muc2* in the proximal colon. (D) Myeloperoxidase activity in the proximal and distal colons of SPF and GF mice inoculated with *Eh*; fold change was compared with untreated SPF mice. (E) Number of filled goblet cells/crypt was blindly counted from the distal and proximal colons in GF and SPF mice. Abx: antibiotics, MPO: myeloperoxidase. n = 6, *P < 0.05, **P < 0.01.

The role microbiota plays in shaping the development of innate host defenses against *Eh* is not known. Based on the results above, microbial dysbiosis induced with Abx in *Muc2*^*+/+*^ and *Muc2*^*-/-*^ specific pathogen-free (SPF) littermates markedly enhanced pro-inflammatory cytokine and mucin secretory responses towards *Eh*. To interrogate the distinct role of microbiota in the development of innate host defenses we quantified *Eh*-induced host responses in colonic loops of germ free (GF) mice. As expected, in response to *Eh*, SPF mice elicited a prompt increase in the expression of the pro-inflammatory cytokines TNF-α and IL-1β mRNA whereas no response was observed in GF mice (**Fig 7A**). This is interesting as enhanced watery secretions were observed in *Eh*-inoculated colonic loops of GF mice (**Fig 1D**). Surprisingly, basal *Math1* and *Muc2* mRNA expressions were very low in GF mice and *Eh* infection did not cause a further decrease as compared to *Eh* inoculated SPF animals (**Fig 7B and 7C**). A similar decrease in myeloperoxidase (MPO) activity, a marker for neutrophils influx into the colon, was also noted in GF mice. This is in contrast to *Eh* in SPF mice that induced 4- and 3-fold increase in MPO activity in the proximal and distal colon, respectively (**Fig 7D**). A dependency for microbiota in *Eh*-induced inflammation was shown by treating SPF mice with Abx that reduced MPO activity to those seen in GF mice in both the proximal and distal colon (**Fig 7D**). As Muc2 and pro-inflammatory cytokine gene expression was associated with increased mucin secretion (**Fig 7A and 7C**), we quantified the number of filled goblet cells (GC) in GF mice. In SPF, there was a significant reduction in filled GC in response to *Eh* as GC are actively releasing mucus in response to *Eh* (see below) whereas in GF mice we did not observe a decrease in filled GC. In fact, GF mice had less numbers of filled GC in the colon (**Fig 7E**).

**Fig 8:**
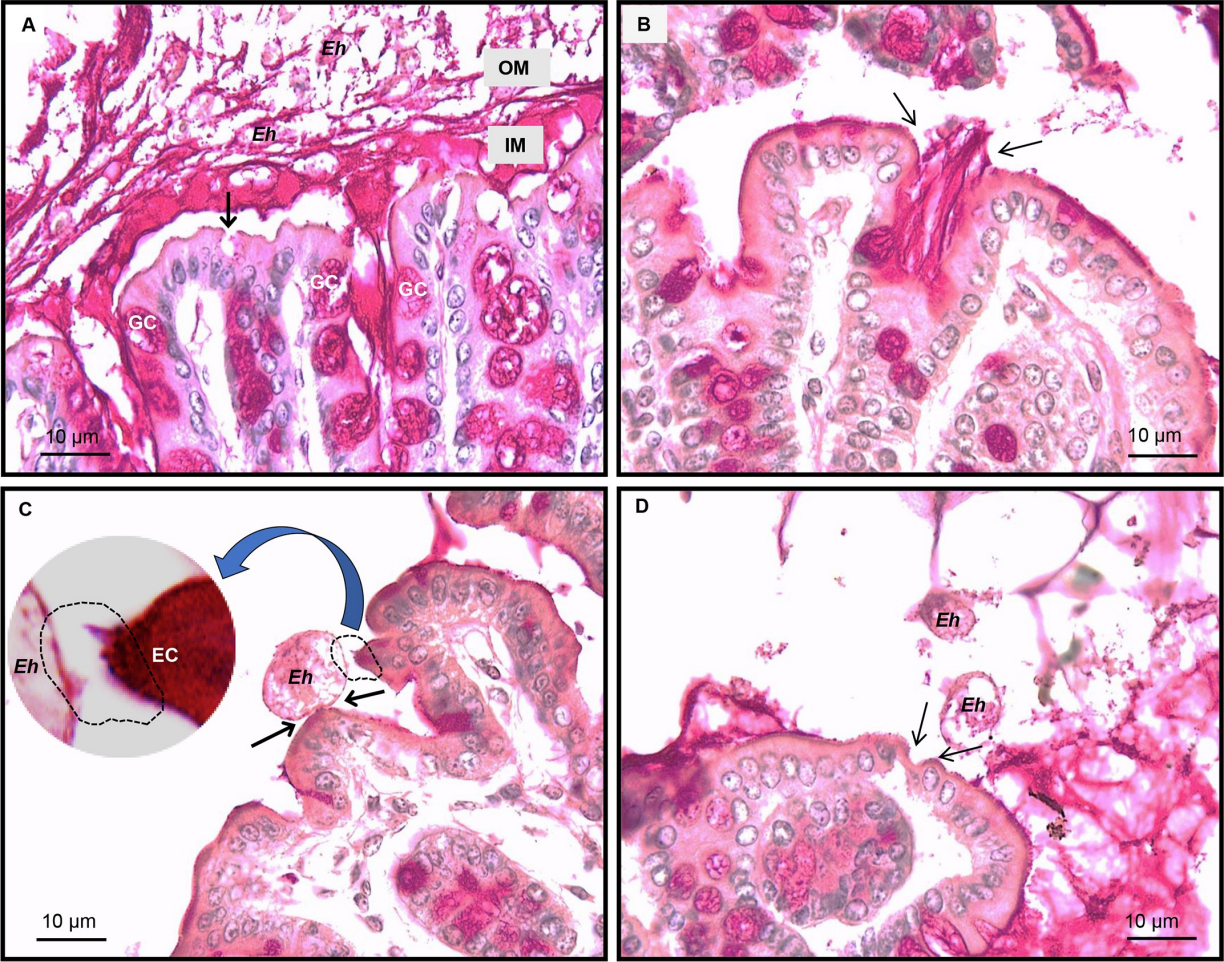
*E*. *histolytica* disrupts the colonic epithelium in germ-free mice. Colonic loops inoculated with *Eh* in specific pathogen-free (SPF) and germ-free (GF) mice were fixed in Carnoy’s solution and stained with periodic acid-Schiff reagent. (A) Representative images of SPF animals inoculated with *Eh* showing thick adherent inner mucus (IM) layer and a loose outer mucus (OM) layer, with a cavitated goblet cell (GC; arrow) due to mucus depletion shown. (B) Intense mucus secretion in response to *Eh* in GF (arrows) mice; note the lower number of goblet cells in the surface epithelium and crypts. (C) *Eh* attaching to GF colonic epithelial cells (EC) (arrows), the epithelial cell on the right appear to be plucked out (inset) by *Eh*. (D) The arrows point at disruption of the single layer of epithelial cells by *Eh* in close proximity to the erosion.

Colonic tissues were fixed in Carnoy’s to preserve the mucus layers and stained with periodic acid-Schiff reagent to visualize mucus, goblet cells and *Eh*. In response to *Eh*, there was hyper secretion of mucus in SPF mice with cavitated (empty, shown by the arrow) GC (**Fig 8A**), thick adherent dense inner mucus (IM) and a loose outer mucus layer (OM) with *Eh* (**Fig 8A**). In GF mice the adherent mucus layer in the proximal colon was patchy with low numbers of GC. Nonetheless, *Eh-*induced intense mucus secretions from GC in the shallow crypts (**Fig 8B**, arrows). Most striking however, unlike SPF were we rarely observe *Eh* in contact with the epithelium, in GF mice *Eh* were occasionally found bound to surface and adjacent epithelial cells (**Fig 8C**; arrows and inset) and at places, showed signs of epithelium erosion in direct contact with *Eh* (**Fig 8D** arrows). Taken together, these results underscore a critical role for microbiota in the development of an effective mucus barrier and host pro-inflammatory cytokine responses in innate host defense against *Eh*.

## Discussion

A major deficiency in our knowledge gap is the relationship between *Eh* and colonic microbiota in parasite colonization, disease pathogenesis and innate host defenses. As microbiota colonizes and utilizes MUC2 mucin substrates as a food source to maintain homeostasis, it stands to reason a delicate balance must exist to sustain asymptomatic *Eh* infections. *Eh* colonizes in/on the MUC2 mucin outer layer and here it interacts freely with colonic microbiota without adverse effects on the host. At present, we do not know the distinct contribution of the microbiota and/or the MUC2 barrier in fortifying innate host defenses against *Eh*. This was the impetus for this study where we interrogated the distinct roles of both colonic microbiota and the mucus barrier in early responses towards *Eh* using colonic loops as a short-term infection model. The major findings of our study revealed that microbial dysbiosis played a critical role in *Eh*-induced water and mucus secretion and pro-inflammatory cytokine responses that was restored following fecal microbial transplants. Moreover, studies in germ free mice revealed that microbiota was critical for shaping the intestinal landscape for the development of goblet cells and formation of an effective mucus barrier and in educating the host pro-inflammatory cytokine responses to limit *Eh* binding and erosion of the surface epithelium.

We have previously shown that *Muc2*^-/-^ are highly susceptibility to *Eh*-induced secretory and pro-inflammatory responses compared to commercially bought WT animals on the same genetic background [16]. In this study, we used *Muc2*^+/+^ and *Muc2*^-/-^ littermates to normalized the microbiota and surprisingly showed no differences in gross pathology scores among the genotypes. This highlights that the use of littermates are essential for microbiota studies as it greatly reduces the variability caused when using animal models in research [22]. Here, the absence of a mucus barrier did not leave *Muc2*^-/-^ mice with a noticeable disadvantage to *Eh* as compared to mucus sufficient littermates, thus demonstrating that the protective role of the mucus barrier is intimately related to the host microbiota. It is well known that an Abx regime provokes alteration in microbial abundance [23]. This particular dysbiotic state is characterized with an increase in facultative anaerobic bacteria within the Enterobacteriaceae family, and has proven to be an indication of a non-homeostatic state in both animal models as well as in important human gastrointestinal diseases [24]. This switch to a more oxygenated luminal environment, could explain the exacerbated reaction towards *Eh*, characterized by an increase in pro-inflammatory cytokines IFN-γ and TNF-α, as well as chemokines MCP-1, KC and MIP-2. *Eh*, despite being a microaerophilic organism, possesses an arsenal of virulence factors to live in the colon [25], but also has various mechanisms that not only protects it, but also allows *Eh* to invade into highly oxygenated environments such as in the case of extra-intestinal amebiasis [26].

In addition to mucin staining, Periodic-acid Shiff (PAS) reagent allowed us to visualize *Eh* and we consistently fail to observe *Eh* in close contact with the epithelium. This is interesting as the host is able to sense *Eh* secreted components and/or the altered environment to mount water and mucus secretions as well as pro-inflammatory cytokine and chemokine responses. Abx treatment alone did not affect basal mucus production or the numbers of filled goblet cells, *Muc2* gene expression or total ^3^H-glycoprotein secretion. Curiously, previous studies have shown that macrolides Abx have an inhibitory effect on mucus production in airways [27] and is used therapeutically in the treatment for chronic obstructive pulmonary disease (COPD), reducing airway goblet cells production of MUC5AC mucin and alleviating COPD symptoms [28]. A similar effect has been described with Muc2 mucin in the gastrointestinal tract, utilising different Abx treatment regimes that reduced the number of goblet cells and *Muc2* gene expression in mice [29], as well as mucus layer thickness [30]. At present, no other studies have reported Abx-induced mucus increase. In our study, we did not observe a reduction but rather, a slight increase in mucus production in Abx-treated mice and could be explained by the Abx regime used.

The mucus layer, secreted by goblet cells, is a key player in maintaining intestinal homeostasis, mainly acting as a barrier and limiting contact between the epithelial cells and any potential hazard contained in the lumen [31,32]. Math1 is a transcription factor that differentiates intestinal stem cells into a secretory lineage, which includes Paneth cells, enteroendocrine cells and goblet cells [33]. Paneth cells are absent in the colon, likewise, enteroendocrine cells are more abundant in the small intestine, but some of them, like L, D and Enterochromaffin cells (EC), can still be found in colon and rectum [34], although, they form only about 1% of the cells in the colon. In our study, we conclude that Math1 activity visualized using Math1^GFP^ had a greater effect on goblet cells than on any other secretory colonic cell lineage. There is a paucity of information on how intestinal pathogens affect the transcription factor Math1. Studies with the nematode parasite *Trichinella spiralis* showed an increase in *Math1* mRNA expression in the small intestine, as well as induction of goblet cell metaplasia when the parasite was present [35], suggesting that Math1 has a protective role in the intestine. DSS-induced colitis in rats had no effect on Math1 activity [36]. Unfortunately, the exact mechanism by which this transcription factor exerts its protective activity is not yet fully described. Although the effect of the Abx cocktail we used was generic, this regime reduced Math1 activity basally in the distal colon. This regional effect could be due to the greater reduction in bacterial load and dysbiosis affecting the distal colon.

An interesting finding was that *Eh* decreased Math1 activity in the proximal colonic loops with a corresponding increase in Math1 activity in the ileum and to a lesser extent in the distal colon. Bacterial translocation in response to *Eh* in colonic loops played a major role in the expression of Math1^GFP^ activity in the ileum and proximal colon. While bacterial translocation have not been studied experimentally with *Eh* infection, translocation of bacteria from the genera *Bacteroides*, *Peptostreptococcus* [37] and *Streptococcus* [3] have been identified in samples from ALA patients. Since the liver is a sterile organ, the presence of commensal bacteria in ALA positive samples indicates that *Eh* infection led to bacterial translocation [3]. In this study, inoculation of *Eh* in colonic loops after 3h increased bacterial translocation in the proximal colon and caused shifts in bacterial populations in the ileum close to the mucosal surface and deep in crypts, this was associated with high MPO activity. Even though we did not detect bacterial translocation in the ileum, there was a significant increase in MPO activity that was not observed in Abx treated or GF animals inoculated with *Eh*. Based on the results of LPS administration it appears that systemic LPS administration accelerated bacterial translocation primarily in the ileum and proximal colon and to a lesser extent, in the distal colon. Commensal bacterial translocation has been reported in *Giardia duodenalis* infection [38].

Studies done in germ free (GF) guinea pigs showed that the presence of microbiota is necessary for *Eh* to express its pathogenicity [9]. Similarly, infection of GF mice with the parasite *G*. *duodenalis* failed to induce the characteristic pathology [39]. Likewise, GF mice infected with the protozoa *Leishmania amazonensis* presented with an innocuous infection and absent immune response towards the parasite [40]. The exact mechanism that explains this phenomenon is not clearly understood, but clearly suggests that microbiota plays a fundamental role in establishing the pathogenicity of these protozoa. Our results are in concordance with previous observations, as *Eh* inoculated in colonic loops of GF mice failed to induce the characteristic pro-inflammatory response in spite of modest water and mucus secretions in the colon. This phenomenon could be due to an undeveloped immune response in GF animals that rendered them with a limited ability to produce cytokines in response to a colonic pathogen. A requirement for cytokines was shown with *Schistosoma mansoni* infection in GF mice, where it is known that TNF-α was required for optimal proliferation of the parasite in the host [41]. The only parasite that showed a worst outcome in GF mice is infection with the protozoa *Trypanosoma cruzi* [42]. The exact mechanisms to explain this altered susceptibility are not known.

Our finding on reduced number of goblet cells correlates with previous reports where a characterization of the colonic epithelium in GF mice showed decreased numbers of goblet cells [43]. Reduction in goblet cells appear to be systemic as it was also observed in paranasal sinuses [44] and in the conjunctiva [45] in these mice. GF mice have a thinner and penetrable intestinal mucus layer [46] and Muc2 monomers with shorter O-glycans [47] compared to conventional SPF mice. Based on this, we hypothesise that the reduction in thickness and changes in the biochemical structure of Muc2 rendered the mucus barrier more susceptible to *Eh* cleavage. It is possible that *Eh* glycosidases and proteases could degrade GF mucus with higher efficiency as they have shorter glycans and absence of commensal microbiota. As *Eh* utilizes microbiota and cleaved Muc2 substrates as its primary source of food, it is tempting to speculate that *Eh* in the GF colon needed to find alternatives nutrient sources, forcing the parasite to move closer to the epithelium. This could explain why we consistently see *Eh* in direct contact with the epithelium with epithelial erosions in the GF colon, a condition we never see in SPF mice.

Taken together, these studies clearly show a requirement for colonic microbiota in forming the first line of innate host defense against *Eh*, independent of the Muc2 mucus layer. Disruption of microbiota with Abx, sensitized animals for exacerbated pro-inflammatory responses and high output water and mucus secretion toward *Eh* that was normalized with fecal microbial transplants. *Eh* infection in the proximal colon increased bacterial translocation and pro-inflammatory cytokine responses that influenced Math1 transcriptional activity of the goblet cell lineage. In the absence of microbiota, *Eh* failed to induce pro-inflammatory responses that together with a dysfunctional mucus layer allowed *Eh* to contact and disrupt the epithelium. This study advances critical roles for both microbiota and the mucus layer in forming layered innate host defenses against *Eh* invasion.

## Materials and methods

### Animals

8 to 10 weeks old *Muc2*^*+/+*^ and *Muc2*^*-/-*^ littermate mice on a C57BL/6 background were used in the experiments. Math1^GFP^ mice (also known as Atoh1^tm4.1Hzo^) [48] were purchased from Jackson laboratory and bred in-house. Germ-free (GF) mice on a C57BL/6 background were purchased from the International Microbiome Centre at the University of Calgary. All animals were housed under specific pathogen-free conditions (SPF) in filter top cages and fed autoclaved food and water *ad libitum*. Throughout the study, animals were closely monitored to ensure healthy conditions; in addition, all experiments adhered to the University of Calgary Animal Care Committee standards.

### Cultivation and harvesting of *E*. *histolytica*

*E*. *histolytica* (HM1:IMSS) trophozoites were cultured in TYI-S-33 medium containing 100 U/ml penicillin/streptomycin at 37°C under axenic conditions. After 72h, logarithmic-growth-phase *Eh* cultures were harvested by chilling on ice for 9 min, pelleted at 200 × g, and washed twice with PBS. Trophozoites were subjected to routine passage through liver of gerbils to maintain high virulence.

### Antibiotic (Abx) treatment

*Muc2*^*+/+*^ and *Muc2*^*-/-*^ littermates were treated with Abx to decrease bacterial load as described previously [49]. Briefly, mice were gavaged every 12h with an Abx cocktail as follow: for the first 3 days mice were gavaged with amphotericin-B (1 mg/kg) to suppress fungal growth. From day 4, ampicillin (1 mg/mL) was added to the drinking water, in addition, mice received orally vancomycin (50 mg/kg), neomycin (100 mg/kg), metronidazole (100 mg/kg) and amphotericin-B (1 mg/kg) for another 14 days. This combination ensures the safe and controlled delivery of Abx to each mouse while having a broad-spectrum effect. Due to its anti-amebic effect, metronidazole was removed from the cocktail during the last 7 days of administration.

### Fecal microbiota transplantation

Fecal microbiota transplantation (FMT) was achieved by collecting 0.1g of mice feces (about 4 fecal pellets), homogenized in 1 mL of sterile phosphate buffered saline (PBS) and centrifuged for 30 seconds at 1000 *x* g. Each mouse was gavaged with 200μl of the obtained supernatant every 48 h for a total of three times.

### Colonic loops an *in vivo* mucin secretion

To quantify mucin secretion *in vivo*, mice were fasted overnight and injected intraperitoneally with 20 μCi of ^3^H-glucosamine (PerkinElmer, Waltham, MA) in PBS for 3h to metabolically label newly synthesized mucin into galactose, *N*-acetyl-glucosamine and *N*-acetyl-*D*-galactosamine in the mucin monomer as described previously [16,50]. Colonic loops were used as a model for short-term infection studies (3h after infection), as described previously [51]. Briefly, *Muc2*^+/+^ and *Muc2*^−/−^ mice were anesthetized with isoflurane inhalant anesthesia (Pharmaceutical Partners of Canada, Richmond Hill, ON). A laparotomy was performed, and the colon was exteriorized and ligated with 3–0 black silk sutures (Ethicon, Somerville, NJ; Peterborough, ON, Canada) at the proximal end and ~2 cm below. Care was taken to keep the mesenteries, blood vessels, and nerves intact. Virulent log-phase *Eh* trophozoites (1 × 10^6^) in 100 μL PBS (pH 7.3) were inoculated into the loop. To quantify secretion of high molecular weight (V_o_) mucin and non-mucin components, secreted ^3^H-labeled glycoproteins were fractionated using a Sepharose 4B columns as described previously [16,50].

### Gross pathology scoring

Gross pathology of colonic loops was assessed on a scale of 1 to 4, as follows: 1, normal colon (uniform thickness, no colon dilation or distension, no blood in luminal contents); 2, minimal damage (visible mucosal thickening and colonic distension, visible mucosal exudates, and expanded loop occupying <50% of the abdominal cavity); 3, extensive damage (thickening of the colonic mucosa, visible dilation of surface blood vessels, colon distension with visible luminal contents, mucosal exudates, and expanded loop occupying 50% of the abdominal cavity); 4, inflamed colon (extensive colon thickening, colon surface with extensive inflamed dilated blood vessels with or without haemorrhage, extensive colon distension with or without visible brown or bloody luminal contents, mucosal exudates under extreme pressure leading to ballooning of the colon, and expanded loop occupying >50% of the abdominal cavity).

### Histology and staining

At the endpoint of the experiments, animals were anesthetised and sacrificed by cervical dislocation and the colon was excised. For histology, colonic tissues were fixed in Carnoy’s solution, and embedded in paraffin blocks. 7μm tissue sections were rehydrated through an ethanol gradient to water and stained with Periodic acid Schiff’s reagent (PAS, Sigma Aldrich Co.) to visualize neutral mucins.

### Quantification of pro-inflammatory cytokines and chemokines

Total RNA was isolated from snap-frozen tissue using the Trizol reagent method (Invitrogen; Life Technologies, Burlington, ON) as per manufacturer’s specifications, and the yield and purity determined by the ratio of absorbance at 260/280nm (NanoDrop, Thermo Scientific). Only samples with a ratio of ~1.8 for DNA and ~2.0 for RNA were considered. cDNA was prepared using a qScript cDNA synthesis kit (Quanta Biosciences). Real-time qPCR was performed using a Rotor Gene 3000 real-time PCR system (Corbett Research). Each reaction mixture contained 100 ng of cDNA, SYBR Green PCR Master Mix (Qiagen) and 1μM of primers. A complete list of the primer sequences and conditions used are listed in Table 1. Results were analyzed using the 2^-ΔΔCT^ methods and expressed as fold changes. Luminal pro-inflammatory cytokines was analyzed using a mouse 31-plex cytokine–chemokine panel (Eve Technologies, Calgary, AB, Canada).

**Table 1:** Primer sequences used for quantitative real-time PCR.

	Name	Sequence 5`3`	Annealing Temp
Murine	IL-1β	Fwd: GCCTCGTGCTGTCGGACCCA Rev: CTGCAGGGTGGGTGTGCCGT	60^o^
	TNF-α	Fwd: ATGAGCACAGAAAGCATGATC Rev: TACAGGCTTGTCACTCGAATT	56^o^
	IFN-γ	Fwd: TCAAGTGGCATAGATGTGGAAGAA Rev: TGGCTCTGCAGGATTTTCATG	54^o^
	Muc2	Fwd: CCCAGAAGGGACTGTGTATG Rev: TGCAGACACACTGCTCACA	56°
	Actin	Fwd: CTACAATGAGCTGCGTGTG Rev: TGGGGTGTTGAAGGTCTC	54^o^
	Math1	Fwd: AAAGGAGGCTGGCAGCAA Rev: TGGTTCAGCCCGTGCAT	58°
	Spdef	Fwd GACTCACACTCAAGGGGCAA Rev: TCAGAAGAGTCGTCCGTCCT	58°
Human	MATH1	Fwd: TGCACTTCTCGACTTTCGAGGACA Rev: AACTTGCCTCATCCGAGTCACTGT	56°
	GAPDH	Fwd: GGATTTGGTCGTATTGGG Rev: GGAAGATGGTGATGGGATT	56°

Murine primers as previously described^17^

### Muc2 fluorescence staining

For visualizing Muc2, 7μm sections of Carnoy´s fixed tissue were incubated with H-300 antibody [1μg/ml] at 4°C overnight and secondary anti-rabbit antibody coupled with Alexa 594 and DAPI (Life Technologies) was used for nuclear counterstain. Tissue sections were visualized using an Olympus FV1000 scanning confocal inverted microscope.

### Math1 expression via non-invasive whole-body imaging *ex vivo*

To detect Math1 associated GFP expression, colons of differently treated Math1^GFP^ mice were surgically removed and imaged *ex vivo* using an In-Vivo Xtreme 4MP-imaging platform (Bruker, Billerica, MA, USA). Colons were positioned horizontally from the proximal to the distal side and imaged with the luminal side facing the camera. The imaging protocol contained two steps: reflectance imaging (2 sec exposure time) and fluorescent imaging with excitation at 470 nm and emission at 535 nm (5 sec exposure time). Binning was kept constant at 2 x 2. Images from the In-Vivo Xtreme were acquired and analyzed using Bruker molecular imaging software MI SE (version 7.1.3.20550). Math1 associated GFP expression in the colon under different treatment conditions was quantified by measuring the mean fluorescence (after background subtraction) in a constant region of interest (ROI). ROI were either defined as whole colon area (proximal to distal part) or split into proximal, median and distal part for quantification. To determine if translocated bacteria caused Math1 expression, a sub lethal dose of LPS (5mg/kg BW) was injected intra peritoneal into Math1^GFP^ animals and the whole gut was surgically removed 24h post treatment. Small intestines were positioned vertically from duodenum to ileum and imaged using an In-Vivo Xtreme 4MP-imaging platform (Bruker, Billerica, MA, USA) as explained above. To avoid autoflorescence derived from diet, mice were fed a non-fluorescent diet (Rodent Diet, AIN-93M, BioServ) for a week before the start of the experiments.

### LS174T cell culture and *Entamoeba histolytica in vitro* assay

Human adenocarcinoma colonic goblet cells (LS174T) were cultured in Dulbecco's Modified Eagle Medium (DMEM) supplemented with 10% fetal bovine serum (FBS), 20 mM HEPES, and 100 U/ml penicillin/streptomycin. Cells were passaged with 0.25% trypsin-EDTA (Thermo) once 90% confluence was reached. For experiments, LS174T cells were seeded in 24-well plates in triplicate at a density of 2.5 × 10^4^ and cultured until a confluent monolayer was formed. To determine if *Eh* directly modulated *MATH1* expression in the absence of microbiota, LS174T cells were exposed to 2.5x10^5^ trophozoites/ml at 37°C for 30 and 60 min. Glutaraldehyde-fixed (2.5% for 15 min and washed twice in PBS) *Eh* was used to determine a requirement for live parasites. LS174T cells were pre-treated with human IL-1β (Peprotech, Cedarlane, Burlington, ON, Canada) at a concentration of 20 ng/mL for 16h to determine if pro-inflammatory cytokines could modulate *MATH-1* expression.

### Intestinal permeability assay

To determine if *Eh* increased intestinal permeability, animals were gavaged with 15mg of fluorescein isothiocyanate (FITC)-dextran (3-5kDa, Sigma Aldrich), dissolved in 100μL of water and colonic loops were performed 2h after. Following *Eh* inoculation in colonic loops for 3h, animals were anaesthetized by isoflurane (Pharmaceutical Partners of Canada, Richmond Hill, ON) and blood was collected by cardiac puncture. Animals were sacrificed by cervical dislocation. Whole blood was allowed to clot in the dark for 3h at room temperature (RT) and centrifuged at 10,000 x *g* for 10 min. Serum was transferred to a clean Eppendorf tube and diluted with an equal volume of PBS. An aliquot of 100μL of each sample was loaded onto a black bottom 96-well plate in duplicate, and fluorescence was determined with a plate reader (absorption 485nm, emission 535nm).

### Bacterial translocation quantification and visualization

For visualizing bacterial translocation animals were gavaged with 200μL of overnight grown culture of *Escherichia coli XEN14* every 24h for 3 days. Colonic loops were then inoculated with *Eh* on *E*. *coli XEN14* infected animals and after 3h, whole gut was surgically removed and imaged *ex vivo* using an *in vivo* Xtreme 4MP-imaging platform, as described previously. Translocated bacterial population was quantified using quantitative PCR method as described previously [52]. For visualization of bacterial translocation in tissues, fluorescence *in situ* hybridization (FISH) was performed as described previously [53]. Briefly, 7 μm sliced Carnoy´s fixed tissue was incubated with the total bacteria probe EUB338 5'-GCT GCC TCC CGT AGG AGT-3'​ [50ng/μL] coupled with Quasar 670 dye at 46°C overnight. FITC coupled-*Ulex europaeus* agglutinin (UEA) was used at [1:1000] to visualize the fucosylated residues in mucins and DAPI [1:1000] (Life Technologies) for nuclear counterstain. Tissue sections were visualized using an Olympus FV1000 scanning confocal inverted microscope.

### Myeloperoxidase activity assay (MPO)

MPO activity in mouse colon samples (50 mg of fresh-frozen tissues) was assessed as a marker for neutrophil influx as previously described [52]. Briefly, tissue was homogenized in 0.5% hexadecyltrimethylammonium bromide in 50 mM phosphate buffer (pH 6.0). Homogenized tissue was freeze-thawed three times, sonicated, and centrifuged (10,000 g for 10 min at 4°C) for collection of clear supernatant. The reaction was initiated by addition of 1 mg/ml dianisidine dihydrochloride (Sigma, St. Louis, MO) and 1% H_2_O_2_, and change in optical density was measured at 450 nm.

### Ethics statement

The Health Sciences Animal Care Committee from the University of Calgary, have examined the animal care and treatment protocol (AC14-0219) and approved the experimental procedures proposed and certifies with the applicant that the care and treatment of animals used was in accordance with the principles outlined in the most recent policies on the “Guide to the Care and Use of Experimental Animals” by The Canadian Council on Animal Care.

### Statistical analysis

Data was analyzed using Graphpad Prism 6 (Graph-Pad Software, San Diego, CA) for all statistical analysis. Treatment groups were compared using analysis of variance (ANOVA) when more than two groups were compared. Student’s t-test was used when only two groups were compared. Statistical significance was assumed at P < 0.05, n = total number of mice per group from two independent experiments. Error bars in all the graphs represent mean ± standard error of the mean (SEM).
